# Patient stratification and identification of adverse event correlations in the space of 1190 drug related adverse events

**DOI:** 10.3389/fphys.2014.00332

**Published:** 2014-09-09

**Authors:** Eva Roitmann, Robert Eriksson, Søren Brunak

**Affiliations:** ^1^Department of Disease Systems Biology, Faculty of Health and Medical Sciences, Novo Nordisk Foundation Center for Protein Research, University of CopenhagenCopenhagen, Denmark; ^2^Department of Systems Biology, Center for Biological Sequence Analysis, Technical University of DenmarkLyngby, Denmark

**Keywords:** adverse events, adverse drugs reactions, electronic medical records, patient stratification, data mining, network analysis

## Abstract

**Purpose:** New pharmacovigilance methods are needed as a consequence of the morbidity caused by drugs. We exploit fine-grained drug related adverse event information extracted by text mining from electronic medical records (EMRs) to stratify patients based on their adverse events and to determine adverse event co-occurrences.

**Methods:** We analyzed the similarity of adverse event profiles of 2347 patients extracted from EMRs from a mental health center in Denmark. The patients were clustered based on their adverse event profiles and the similarities were presented as a network. The set of adverse events in each main patient cluster was evaluated. Co-occurrences of adverse events in patients (*p*-value < 0.01) were identified and presented as well.

**Results:** We found that each cluster of patients typically had a most distinguishing adverse event. Examination of the co-occurrences of adverse events in patients led to the identification of potentially interesting adverse event correlations that may be further investigated as well as provide further patient stratification opportunities.

**Conclusions:** We have demonstrated the feasibility of a novel approach in pharmacovigilance to stratify patients based on fine-grained adverse event profiles, which also makes it possible to identify adverse event correlations. Used on larger data sets, this data-driven method has the potential to reveal unknown patterns concerning adverse event occurrences.

## Introduction

Adverse drug reactions (ADRs) have been reported to be directly responsible for more than 5% of all hospitalizations (Pirmohamed et al., 2004; Kongkaew et al., 2008) and add considerably to the morbidity of patients and the cost of healthcare (Rottenkolber et al., 2012). Therefore, new methods for analyzing adverse event (AE) trends and for identifying causal relationships to determine ADRs are of considerable importance (Wang et al., 2009; Coloma et al., 2012; Harpaz et al., 2012, 2013; Eriksson et al., 2013). Such knowledge is essential for providing more informed medical care and thereby in particular preventing ADRs (Landmark and Johannessen, 2012; Yusof and Hua, 2012).

We have developed a method for analyzing AEs based on a data mining approach for elucidating patterns of patient-specific AE occurrences. The approach exploits phenotypic information in the clinical narratives of electronic medical records (EMRs). There is currently major focus on employing EMRs for pharmacovigilance due to the limited scope of clinical trials and the need for complementing spontaneous reporting systems (McClellan, 2007; Coloma et al., 2012; Harpaz et al., 2012; Eriksson et al., 2014).

Clinical trials are limited in size and characteristics of the patient cohorts included in the studies, and further limited by the duration of the trial (Harpaz et al., 2012). Spontaneous reporting systems, the current principal method for pharmacovigilance (Avery et al., 2011; Harpaz et al., 2012), are inherently biased and suffer from incomplete data, under-reporting of AEs (Hazell and Shakir, 2006), and over-reporting of ADRs that receive media attention (Harpaz et al., 2012). Moreover, the traditional methods within pharmacovigilance have in several cases led to the discovery of critical ADRs only after several years on the market for a given drug (Kälviäinen and Nousiainen, 2001; McClellan, 2007). As the implementation of EMRs is developing rapidly (Kierkegaard, 2011; Hatton et al., 2012), large amounts of phenotypic data are systematically and automatically recorded in a population-wide fashion. Data mining of information contained in EMRs has the potential to identify ADRs earlier (Brownstein et al., 2007), improve prevalence measurements of AEs (Coloma et al., 2011; LePendu et al., 2013), and to create the basis for obtaining better understanding of AEs, which may help guide clinical decision making to improve patient safety (Jensen et al., 2012).

EMRs typically contain both structured data and clinical narratives, where the latter represent free text authored by healthcare staff. The use of EMRs for pharmacovigilance research has so far mainly been based on structured data (Coloma et al., 2011). However, it has been suggested that up to 90% of AEs are recorded only in the clinical narratives (Classen et al., 2011; Roque et al., 2011; Haerian et al., 2012). Hence, more AEs will be captured through extraction by natural language processing (Meystre et al., 2008; Eriksson et al., 2013; LePendu et al., 2013).

Specialized ADR terminologies and dictionaries such as the WHO Adverse Reactions Terminology (WHO-ART) (The Uppsala Monitoring Centre, 2014) and the Medical Dictionary for Regulatory Activities (MedDRA) (Brown et al., 1999) have unfortunately not been translated into all languages, including Danish or any other Nordic language. When performing AE extraction in a language like Danish it is therefore relevant and necessary to construct novel, aggregated dictionaries to handle comprehensive, specific AEs and capture more fine-grained information in the clinical narratives (Eriksson et al., 2013). While most research focused on a few selected AEs or drugs (Trifirò et al., 2009; Wang et al., 2009; Haerian et al., 2012), we aimed to analyze the full spectrum of AEs described in EMRs. Analyzing all AEs present in EMRs additionally reflects actual drug use in heterogeneous populations. Several traditional methods analyzing AEs rely on spontaneous reporting systems such as the US FDA Adverse Event Reporting System (FAERS), the European Economic Area collaboration Eudravigilance and the WHO VigiBase. However, such reporting systems may suffer from over-reporting of ADRs for drugs with known and published ADRs, under-reporting of unknown ADRs (the causal link is not identified), and otherwise missing or incomplete data (Harpaz et al., 2012). As there is no preconception in EMRs around which AEs to expect, the resulting information is likely to be less biased compared to the information gathered in large databases of spontaneous reporting systems. This possibly allows discovering new and unexpected findings concerning AEs.

We have developed a patient-stratification scheme based on a data mining approach for elucidating patterns of patient-specific AE occurrences. The extracted AEs are subsequently analyzed in terms of their co-occurrences across a patient cohort.

The strategy presented here for exploring AEs relies partially on network analysis, which describes topological and quantitative relationships between either AEs, patients, or drugs. In recent years, the use of networks has increased in biomedical research (Barabási et al., 2011) to explore complex interrelated systems e.g., to identify novel disease proteins or genes associated with a given phenotype (Lage et al., 2007, 2010) as well as proteins related to ADRs (Huang et al., 2011; Chen et al., 2013). Networks have also been used extensively to better understand the structure of diseasomes, including co-morbidity analyses (Goh et al., 2007; Hidalgo et al., 2009; Barabási et al., 2011; Roque et al., 2011). Similarly, in this work we use networks as they represent an excellent method of exploring and understanding data sets of extreme dimensionality, with thousands of AEs and millions of potential AE-AE pairs. We use the technique to study and provide a cohort-wide overview of the co-occurrences of AEs in same individuals to reveal potentially interesting interdependencies between AEs. Patients may be stratified according to their AE profiles and the AE-AE correlations the patients encounter.

The method presented here has been employed on EMRs from a Danish mental health center. Many patients with psychiatric disorders need to strike a balance between efficacy and safety. This is because the disorders are so severe that a fair number of ADRs are accepted during treatment (Bender et al., 2004; Muench and Hamer, 2010; Jain et al., 2011; Landmark and Johannessen, 2012). As the use of e.g., neuroleptics is nearly always life long, the importance of pharmacovigilance within psychiatry is emphasized. We present a method, which scales to larger data sets and patient populations. The method is also well-suited for further integration of findings with genetic, proteomic, and pathway data as well as existing chemoinformatics knowledge relating to the drugs that cause the ADRs when administered to patients. A possible way to perform such analysis is to map AEs to OMIM and create protein-protein interaction networks, in a similar way as previously described (Roque et al., 2011).

## Methods

### Ethics statement

AE co-occurrences were analyzed based on de-identified data. The project was ethically approved by the Danish National Board of Health (J. nr. 7-604-04-2/33/EHE).

### Patient corpus

The EMRs employed in this study are from a Danish mental health center and hold data from 6011 patients collected between 1998 and 2010, and therefore contain extensive longitudinal data on many patients. Both structured and unstructured EMR data were used. The clinical narratives were used for extracting AEs by text mining, while the structured data was used for obtaining precise information concerning drug identities (using the Anatomical Therapeutic Chemical, ATC, system), drug dosages, prescription intervals, and diagnoses (using International Classification of Diseases, 10th revision, ICD10). It was identified that 3394 patients were prescribed at least one drug while 2347 patients had at least one AE. The gender ratio for patients consuming at least one drug was 1:2 female to male, while it is 2:3 for patients that have at least one AE. The prescribed number of drugs to the population with at least one AE was at an average of 15.4 with standard deviation of 9.4 per patient. The mental health center investigates and treats patients who suffer from severe mental illnesses, and the center also has a ward for forensic psychiatry. It is therefore not representative of a general population. The disease distributions have previously been described elsewhere (Roque et al., 2011). Hence, it is important to interpret the results in the appropriate context of a mentally ill population and with forensic hospitalizations. However, the method presented is not in any way limited to this particular indication area.

### Extraction of adverse events

The AE text mining extraction from the clinical narratives is elsewhere described in detail (Eriksson et al., 2013, 2014). The method relied on a Danish dictionary of AEs constructed based on the undesirable effects section from the summary of product characteristics of 7446 drugs marketed in Denmark. In all, 21,342 uniquely spelled ADRs from the summary of product characteristics were used to construct an initial ADR dictionary, which was further condensed in different ways. ADR terms were coordinated post-extraction based on a class system and synonymous terms or terms with the same medical implementation were merged. The extracted terms were filtered in order to find only true possibly drug related AEs e.g., by eliminating (i) negated concepts or mentioning of other subjects, (ii) information given to the patient about possible undesirable effects or events that the patient previously experienced, (iii) symptoms that are an indication for the drug, (iv) potential ADRs that are from sentences with two or more drugs, as this was likely to be medical history or information to the patient, and (v) pre-existing conditions that the patients had prior to intake of the drug and experienced during treatment. A temporal data mining approach was used that combined the terms with information from structured data of prescription period and prescribed drug dosage (Eriksson et al., 2014). The approach identified 75% of the total possible drug related adverse events (recall/sensitivity) and in 95% of the cases the identified AEs were actual possible drug related adverse events (precision/positive predictive value). The validation was carried out by comparing with manual inspection of a sample of 200 randomly selected patient notes (Eriksson et al., 2014). The dictionary employed in this research underwent slight modifications to become the final version described by Eriksson et al.

### Overview of methods for data analysis

The methods presented in this paper are summarized in Table 1. Further detail is provided in the following sections.

**Table 1:** **Overview of methods used to analyze AEs**.

	**Type of analysis**	**Vector values**	**Associations**	**Clustering**	**Filtering**	**Visualization**
Patient stratification	Clustering	Tf-idf per patient	Cosine dissimilarity	Hierarchical clustering	Threshold	Network
	Analyzing cluster characteristics	Tf-idf per cluster - fraction of vector sum	Euclidian distance	Hierarchical clustering	n/a	Heat map
AE co-occurrences	Finding co-occurrences with method 1	Binary	Co-occurrence score	n/a	Fisher's exact test Benjamini and Hochberg	Network
	Finding co-occurrences with method 2	Binary	Weighted edges	n/a	Multiscale Backbone	Network

### Patient stratification

Patients were represented by vectors in a space of 1190 AE dimensions. The values for each AE in the vector were term-frequency inverse document frequency (tf-idf) weighted values in order to correct for how strongly a patient was associated with a given AE. The tf-idf is a statistical technique to determine how common a term is to a document in a corpus (Robertson and Spärck Jones, 1976). The method takes into account how many times the term appears in a patient's medical history (term frequency) and the prevalence of the term in the whole corpus (document frequency). In this study, a normalized value of term frequency was calculated by dividing the number of times a specific AE term appears in a patient's medical history with the number of times all AE terms appear. This was done to prevent bias toward patients with longer clinical narratives. The tf-idf is defined by:

$$\text{tfidf}=\frac{f}{F}\cdot \text{ln}\left(\frac{N}{n}\right)$$

where *f* is the number of times a given AE term appears in the EMR, *F* is the number of times all AE terms appear in the patient's medical history, *N* is the total number of patients, and *n* is the number of patients that have the specific AE.

The patients were stratified using the cosine dissimilarity measure to quantify the distance between their vectors in the AE space. The cosine dissimilarity is one minus the cosine of the angle between the two vectors:

$$\text{cosine dissimilarity}=1-\cos\left(\theta \right)=1-\frac{a\cdot b}{\text{||}a\text{|| ||}b\text{||}}$$

The cosine dissimilarity measure has the advantage of being independent of the length of the vector, meaning that in spite of two patients not having the same number of AEs they may still be compared meaningfully. This method is a measure commonly used in the literature when handling phenotype vectors (Lage et al., 2007; Chan et al., 2011; Roque et al., 2011).

Patients were clustered by average linkage hierarchical clustering. A cutoff was set at a cosine dissimilarity value of 0.6 after manual inspection of the clustering dendrogram. This yielded 720 clusters in total where 45 contained 10 patients or more accounting for 976 patients.

In order to investigate the clinical characteristics of the patients in the clusters three new tf-idf vectors were constructed per cluster containing (1) the AEs, (2) the drugs associated to the AEs, and (3) the diagnoses. The tf-idf vectors were calculated for the merged EMRs of all the patients in a cluster. In order to better compare values between vectors a characteristic scale with values from 0 to 1 was introduced, which was the fraction that each AE composed out of the vector sum.

### Co-occurrence of adverse events

Every distance measure has a bias that over- or under-estimates relationships between rare or prevalent elements. Results should always be interpreted bearing in mind that the method is decisive for the results observed. For the purpose of ensuring that results of detected co-occurrences in AEs are consistent independently of the method used, two methods were used for evaluating associations between elements, namely finding a co-occurrence score and calculating weighted edges. In total there were 707,455 ((1190^2^–1190)/2) possible AE pairs and the two mentioned methods identified the most relevant ones.

#### Co-occurrence score

First, a co-occurrence score was calculated reflecting how often a pair of AEs occurs in a patient with regard to what could be expected, a common method for quantifying co-morbidities (Hidalgo et al., 2009; Roque et al., 2011). The number of patients that are affected by both AE A and B, *Obs* = *n_AB_*, is divided by the expected number of observations, which is the product of the number of patients that are affected by A and B divided by the total number of patients *Expt* = *n_A_n_B_/n_tot_*. To favor pairs of low prevalence AEs less, a pseudo-count of 1 was added to the nominator and denominator. The co-occurrence score is given by:

$$co-occurence\;score=\log_{2}\left(\frac{Obs+1}{Expt+1}\right)=\log_{2}\left(\frac{n_{AB}+1}{\frac{n_{A}n_{B}}{n_{tot}}+1}\right)$$

Second, the most significantly associated AE pairs were found by calculating a *p*-value for each AE pair by Fisher's exact test. The test was performed for each pair of AEs (A and B) on four groups of patients: A and B, A not B, B not A, and not A not B divided according to whether the patients were affected by the AEs. We used the Benjamini and Hochberg method to correct for multiple testing (Benjamini and Hochberg, 1995). The *p*-value s from the Fisher's exact test were ordered and each value was multiplied by the number of tests and divided by the rank that the AE pair had in the ordered list. Only pairs with a corrected *p*-value below a cutoff of 0.01 were selected.

#### Weighted edges

The methods employed in this second analysis are described in detail elsewhere (Barrat et al., 2004; Serrano et al., 2009) and are briefly presented here. The associations between AEs were based on which patients they affect. The strength of the association between AEs was given by the weighted edge, which is the sum of the number of patients that had both of the compared AEs normalized by the number of AEs that affected the given patient. The weighted edge between AE *i* and AE *j* was defined as:

$$weighted\;edge_{ij}=\sum_{p}\frac{\delta_{i}^{p}\delta_{j}^{p}}{n_{p}-1}\;if\;i\neq j,\;WE_{ij}=0$$

where the index *p* runs over all patients, *n_p_* is the number of AEs that affect the patient, and δ*^p^_i_* is 1 if the patient has AE *i* and 0 if the patient does not have AE *i*.

For identifying the most significant associations of out the possible AE pairs, the multiscale backbone was extracted from the network. This approach tests every weighted edge value from AE *i* against an expected value produced by a random assignment from a uniform distribution. The formula used to calculate the *p*-value of an edge from AE *i* to AE *j* is given by:

$$\alpha_{ij}=1-\left(k-1\right)*\int_{0}^{p_{ij}}{\left(1-x\right)}^{k-2}dx$$

where *k* is number of edges from AE *i*, *x* is a particular value that the weight can assume, *p_ij_* is the weighted edge from AE *i* to AE *j*, normalized by dividing with the sum of all edges from AE*i*: *p_ij_*= *weighted edge_ij_*/∑_*j*_weighted edge_ij_ (the fraction that this edge represents out of the sum of all edges from AE *i*). If the *p*-value was below a significance level α of 0.01, the association between the two AEs was significant.

#### AE co-occurrence score visualization

To ease visualization a rough division of AEs into anatomical areas was performed. As the terms cannot be directly linked to the ICD10 system, because the precise AE terms do not appear in the ICD10 dictionary, this division was performed manually and was merely inspired by the ICD10 classification.

## Results

### Statistics of the data

**Figure 1:**
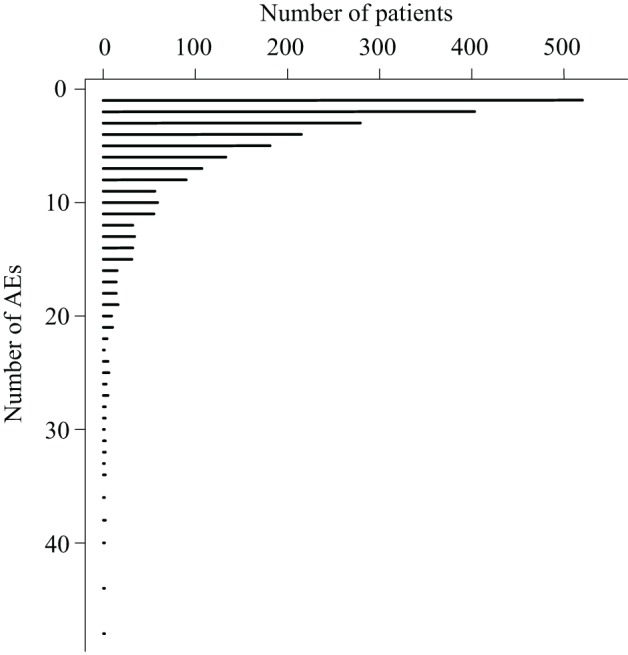
**The number patients that are affected by a certain number of adverse events**.

**Figure 2:**
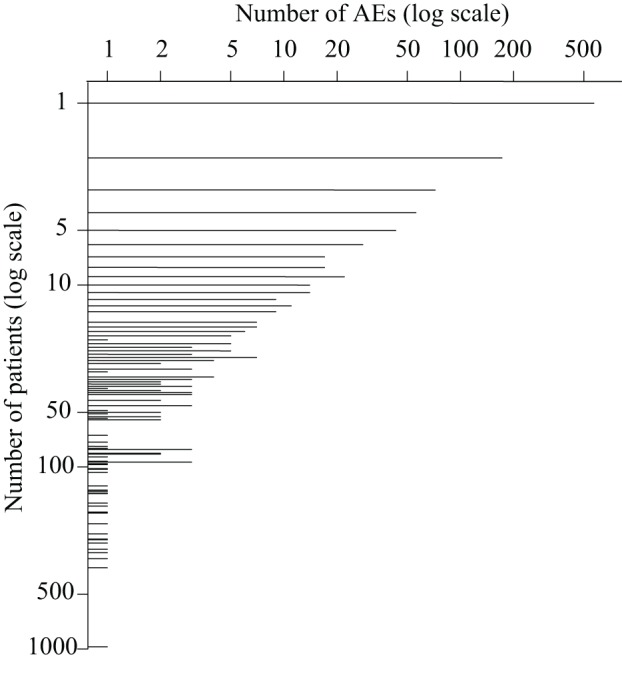
**The number of adverse events that affect a certain number of patients**.

When applying the text mining pipeline on the clinical narratives it was determined that the EMRs contained 2347 patients affected by at least one AE. Taken together, these patients had 1190 unique AEs, on average 5.2 AEs per patient (Figure 1), and 10.3 patients per AE (Figure 2). The maximal number of AEs per patient was 48, while 22% of patients were affected by only one AE identified by text mining. Moreover, 48% of AEs affected only one patient.

### Patient stratification

**Figure 3:**
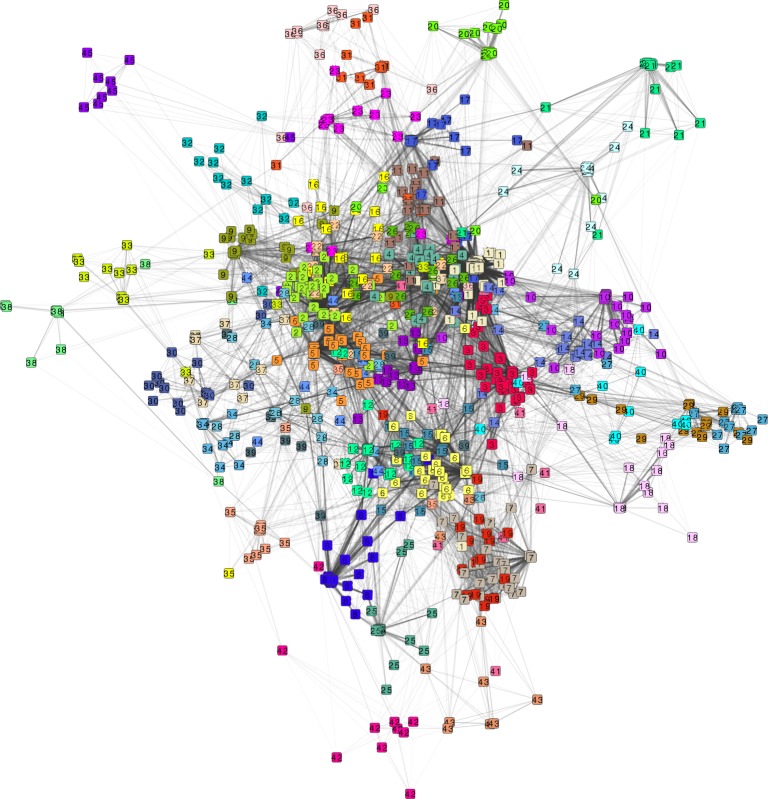
**Network of 976 patients and 25,804 patient pair associations based on the patient adverse event profile**. Each node represents a patient and the links between the patients are associations with a cosine dissimilarity value of less than 0.6. Node color denotes cluster membership as determined by hierarchical clustering with a cutoff at 0.6. Shown are only clusters with 10 or more patients, yielding 45 clusters.

Each patient was represented by a vector in the space of AEs, reflecting the AE profile of the patient. The network shown in Figure 3 is a spatial representation of the associations between patients, reflecting to what extent their AE profiles display similarity. Patients are colored according to which cluster they belong, following the result of the hierarchical clustering. Only clusters with 10 or more patients (45 clusters in total) have been included in the figure, which yielded a network of 976 patients and 45 clusters. The clusters with the smallest numbers are the largest clusters, for example cluster 1 has 122 patients and cluster 45, 10 patients.

#### Cluster adverse events

**Figure 4:**
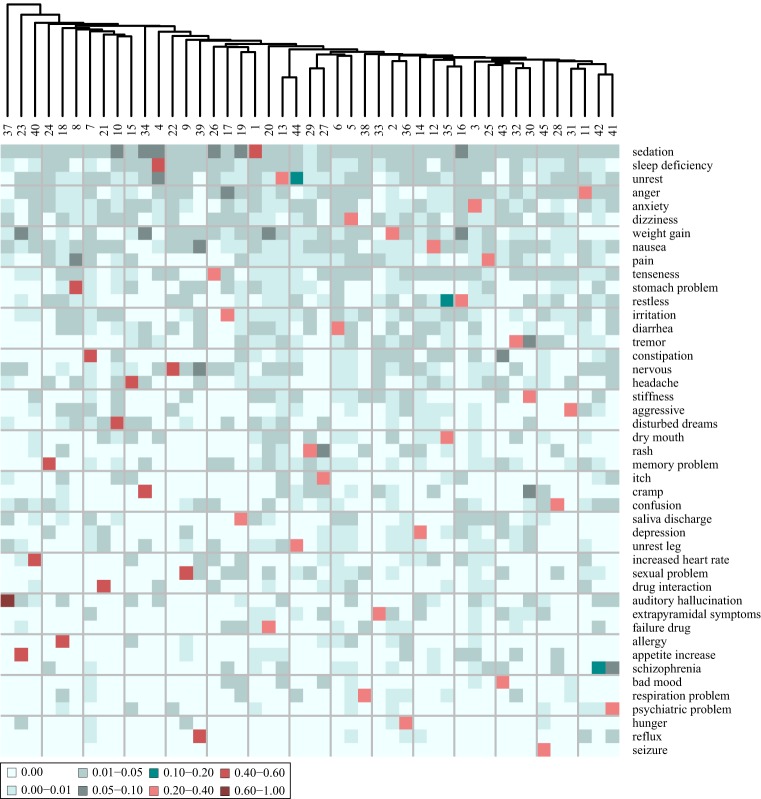
**Heat map of the adverse event (AE) composition of 45 clusters, based on clustering of the AE profiles of the patients**. The values, indicated by color, display the fraction that each AE represents out of the total tf-idf vector sum. Shown are only the 45 AEs that comprise most distinguishing AEs of each cluster. The AEs are ordered according to their prevalence in the entire patient corpus, the most prevalent at the top.

The nature of the patient stratification is further illustrated when coupled to the cluster characteristics in terms of which AEs the patients of each cluster have (Figure 4).

From Figure 4, it is evident that all clusters have one AE that stands out as the most distinguishing AE for this cluster. None of the 45 clusters share the same most distinguishing AE. The most distinguishing AE constitutes more than 20% of the sum of the tf-idf values in the AE vector of each cluster except cluster 42. For example “sedation” is the most distinguishing AE of the patients in cluster 1 as it accounts for 40% of the tf-idf vector sum. The AEs that are the most distinguishing for the patients in the clusters are also the most prevalent AEs at the mental health center. This can be concluded from the observation that the AEs on the heat map of Figure 4 are ordered according to prevalence and the first 18 on the list (from “sedation” to “headache”) are also the 18 most prevalent AEs at the center. Patients at the periphery of the network have more distinct AE profiles than patients in the center of the network. In general, Figure 3 shows that the largest clusters (clusters 1–6) gather in the center of the network while clusters with fewer patients are at the periphery (e.g., clusters 38, 42, and 45). This is likely because the most distinguishing AEs of the largest clusters are in general also the most prevalent ones and many patients from other clusters are also affected by those AEs.

In addition to the most distinguishing AE, many of the clusters also have a second AE that represents a fairly large fraction (5–10%) of the tf-idf vector sum. This indicates that there is some level of systematic co-occurrence of AEs. For example, the most distinguishing AE of the patients of cluster 23 is “weight gain” accounting for 58% of the tf-idf vector sum while “appetite increase” constitutes 5.4%. Also, the patients of cluster 30 share many AEs: “stiffness” 28.3%, “tremor” 5.7%, and “cramp” 5.1%. The patients of cluster 35 have “dry mouth” as the most distinguishing AE accounting for 30% of the tf-idf vector sum while “restless” constitutes 11%. These trends are further investigated through a co-occurrence analysis described below.

**Figure 5:**
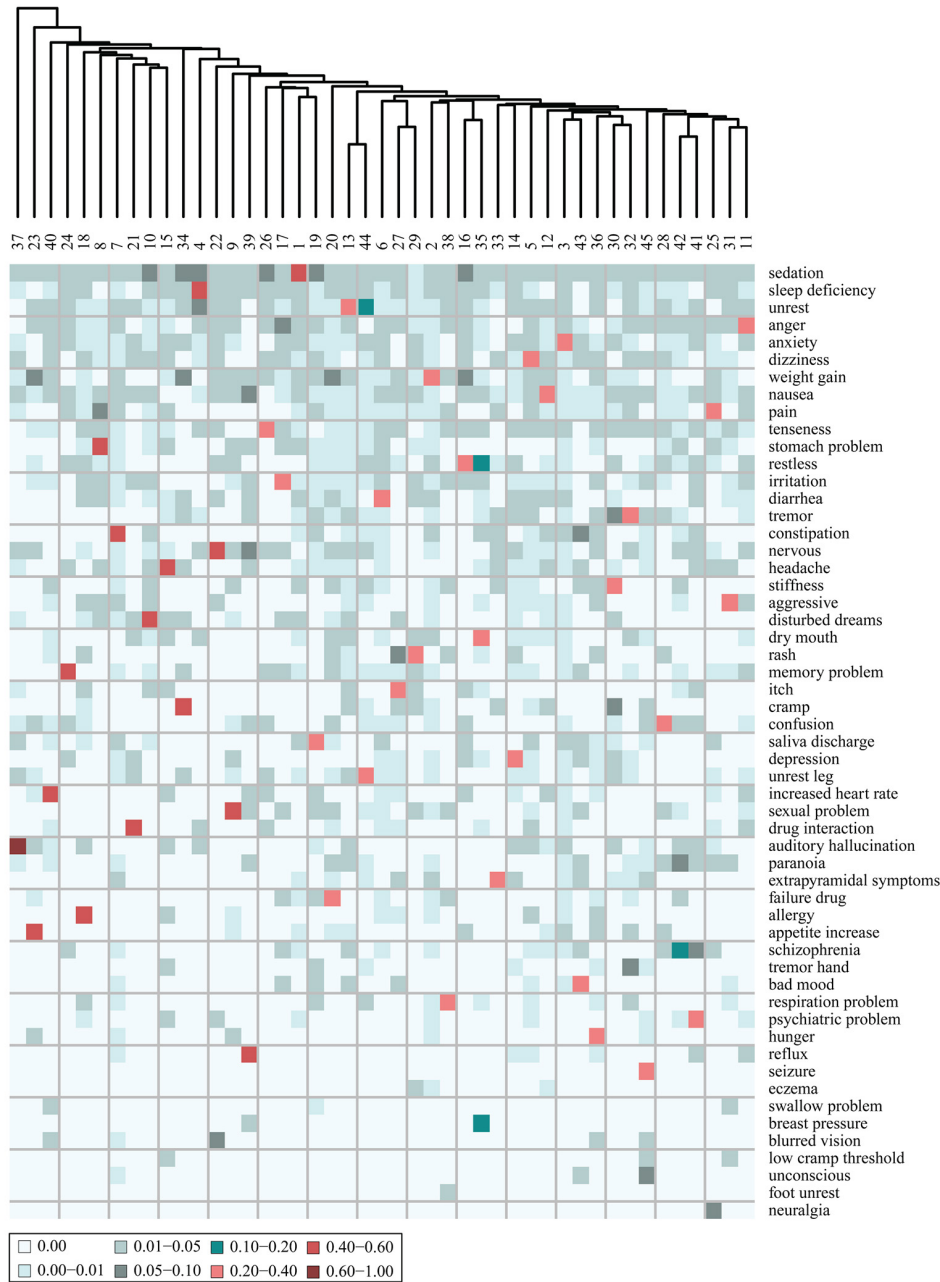
**Heat map of the adverse event (AE) composition of 45 clusters of patients, stratified based on the AE profiles of the patients**. The values are the fraction that each AE represents out of the tf-idf vector sum. Shown are the 55 AEs that comprise the two most distinguishing AEs of each cluster. The AEs are ordered according to their prevalence in the patient corpus, the most prevalent at the top.

The AEs of each cluster were analyzed further by including not only the most distinguishing AE of each cluster but also the second most distinguishing AE, presented in a heat map in Figure 5. It is evident that for all clusters, the most distinguishing AE constitutes a much larger fraction of the tf-idf vector sum than the second most (on average 8.2 times larger). It can thereby be inferred that the stratification is effective and it might be reasonable to consider the patients in a cluster as representatives of the most distinguishing AE of this cluster. Moreover, only 10 new AEs are added to the list of AEs, which indicates that only 10 of the clusters have a second most distinguishing AE that is not the most distinguishing AE of another cluster.

#### Cluster specific drugs

**Figure 6:**
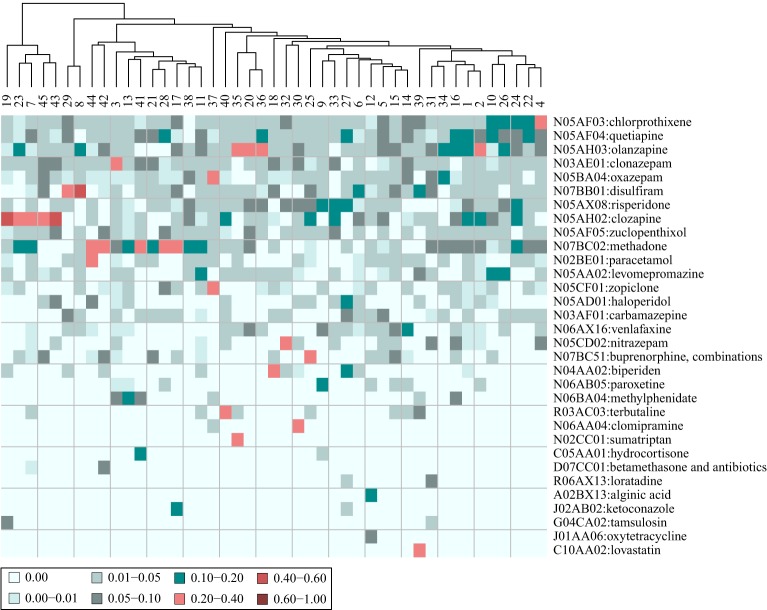
**Heat map of the composition of drugs that are assumed to cause the adverse events (AEs) affecting the patients in 45 clusters**. The patients are stratified based on their AE profiles. The values are the fraction that each drug represents out of the tf-idf vector sum. Shown are the 32 drugs that comprise the two most distinguishing drugs for each cluster. The patient clusters are further clustered on the basis of their drug profiles.

Taking the most distinguishing drug that caused the AEs that affect the patients in each of the 45 clusters (i.e., the drug with the largest tf-idf fraction) yielded a vector of 23 ATC level 5 drugs. Thus, some drugs are the main cause for the AEs of several clusters. When including the two largest tf-idf fractions, a vector of 32 ATC level 5 drugs was generated (visualized in Figure 6). Including the five largest tf-idf fractions, generated a vector of 70 ATC level 5 drugs.

When inspecting Figure 6 it can be observed that most of the drugs that caused the AEs of the different clusters are placed in the ATC chapter N, Nervous system (72%). On one hand this reflects that these drugs were the most prevalent at this particular mental health center and on the other it could be inferred that these drugs typically have several AEs. The clustering based on tf-idf fraction vectors in ATC level 5 drug space in Figure 6, shows that some clusters have a similar profile of drugs that cause the AEs of the patients in the cluster. For example, there is a group of clusters (group A: 19, 23, 7, 45, and 43) whose AEs were mainly correlated with clozapine (N05AH02), while the AEs of another group of clusters (group B: 44, 42, 3, 13, 41, 21, 28, 17, 38, and 11) were mainly caused by methadone (N07BC02). The most distinguishing AEs of these groups of clusters (group A: “saliva discharge,” “appetite increase,” “constipation,” “seizure,” and “bad mood,” and group B: “unrest leg,” “schizophrenia,” “anxiety,” “unrest,” “psychiatric problem,” “drug interaction,” “confusion,” “irritation,” “respiration problem,” and “anger”) may therefore be related as they are caused to a large extent by the same drugs.

Some clusters have two or three drugs that are the main culprits for the AEs of the patients in these clusters. For example, for cluster 37, with “auditory hallucination” as the most distinguishing AE, oxazepam (N05BA04) comprises 34% of the vector sum, while zopiclone (N05CF01) comprises 21%, and for cluster 35, with “dry mouth” as the most distinguishing AE, olanzapine (N05AH03) comprises 22% of the vector sum, while sumatriptan (N02CC01) comprises 27%. Hence, the AEs of the patients with prescription of these drugs may be a result of the combination of the drugs. However, in order to conclude this, additional in depth analyses should be performed. In general, the value for the most distinguishing drug for each cluster was only 2.2 times larger than the value of the second most distinguishing one (compared to 8.2 with the AEs of the clusters). This further accentuates that several different drugs are the causes for the AEs of the patients in the different clusters.

#### Cluster specific diagnoses

**Figure 7:**
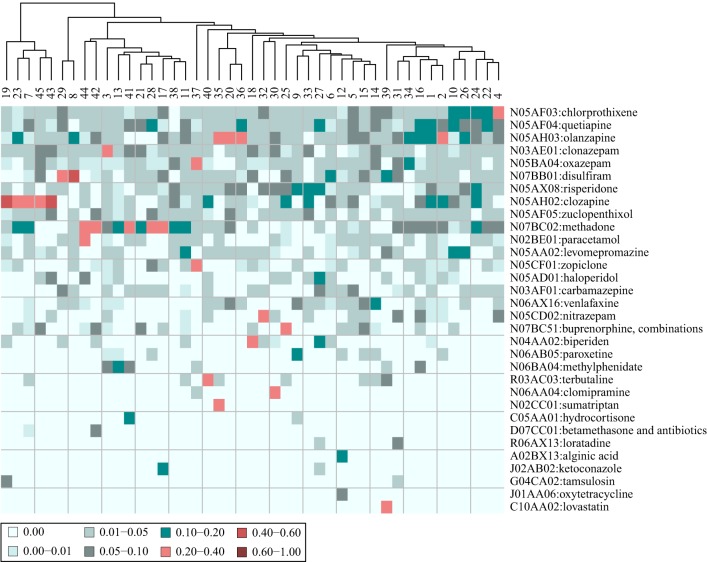
**Heat map of the composition of diagnoses characterizing the patients in 45 clusters**. The patients are stratified based on their adverse event profiles. The values are the fraction that each drug represents out of the tf-idf vector sum. Shown are the 44 diagnoses (in collapsed ICD 10 codes) that comprise the five most distinguishing diagnoses for each cluster. The patient clusters are further clustered on the basis of their diagnose profiles.

Taking the most distinguishing diagnose of patients in each cluster (i.e., the diagnosis with the largest tf-idf fraction) yielded a vector of 30 ICD10 level 3 coded diagnoses. This means that for some of the clusters, the most distinguishing diagnosis is the same. When including the second largest tf-idf fractions, a vector of 44 ICD10 level 3 coded diagnoses was produced, which is visualized in a heat map in Figure 7. Including the five largest tf-idf fractions yielded a vector of 74 ICD10 level 3 coded diagnoses.

In general, the most distinguishing diagnose for a cluster did not stand out compared to the most distinguishing AE for each cluster, as the fraction it composed out of the vector sum was only 1.7 times larger than the second most distinguishing diagnose. In all, 9 clusters had a most distinguishing diagnose that compose more than 10% of the vector sum, while 10 clusters had no diagnose that compose more than 5% of the tf-idf fraction sum. Additionally, 14 clusters had two diagnoses that both comprised a fraction of 5–10%.

The clustering based on tf-idf fraction vectors in ICD10 level 3 coded diagnosis space in Figure 7, shows that most clusters ended up in one large cluster while a few clusters in the left of the heat map were markedly different. This indicates that the patients tended to have many of the same diagnoses across clusters.

When extracting the diagnoses of the patients from the clinical narratives, the diagnoses that are possible AEs were not filtered out. Hence, as a result of the method used, it could be that the AEs that affected the patients were similar to the diagnoses that the patients in the clusters had. This was only the case for three clusters, namely cluster 45, where the patients had an epilepsy diagnose and experienced seizures as an AE, cluster 38, where patients had a pneumonia diagnose and respiration problems as an AE, and cluster 41 with patients that had pervasive developmental disorders and had psychiatric problems as an AE. Thus, the diagnoses and the AEs of the patients of the clusters were in general not similar.

An unexpected correlation between diagnoses and AEs of the patients in the clusters might indicate a causative factor, meaning that patients with a certain diagnose are more likely to experience a certain AE. For example cluster 35 had patients diagnosed with migraine that experienced “dry mouth” as an AE, cluster 43 had patients with dental caries that experienced “bad mood” as an AE, and cluster 32 had patients with a psoriasis diagnosis who had “tremor” as an AE.

### Co-occurrence of adverse events

#### Co-occurrence score network

**Figure 8:**
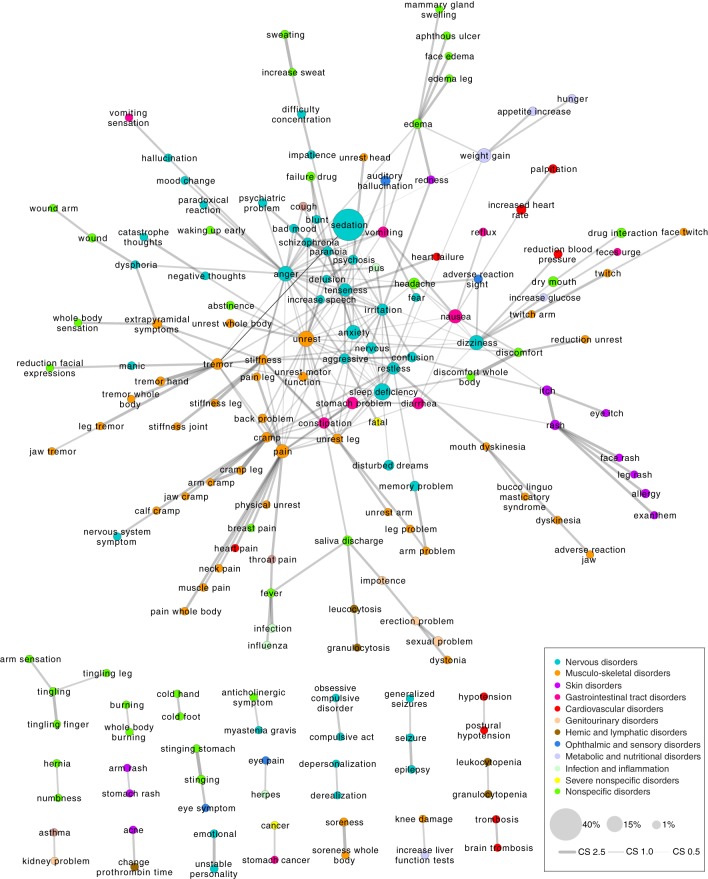
**Network of adverse event (AE) associations based on which patients the AEs affect**. Shown are only the significant co-occurrence score values as determined by Fisher's exact test with a false discovery rate control at a significance level of 0.01. In this network, there are 173 AEs and 262 edges. The nodes are colored according to the anatomical class they have been assigned to (inspired by the International Classification of Diseases version 10 system). Node size reflects the fraction of patients out of the entire patient population that are affected by a given AE.

The co-occurrence of AEs analysis yielded an AE-AE network comprising 173 AEs and 262 significant co-occurrence score value associations (see Figure 8). The figure contains one large interconnected network and a number of smaller clusters separated from the large network. The AEs are colored according to the anatomical area they relate to.

It can be observed that AEs related to the same anatomical area tend to gather in the same regions of the network e.g., the nervous disorders (cyan) are spatially close to each other and the same applies to the musculo-skeletal disorders (orange) and skin disorders (purple). This indicates for example that patients with skin AEs tend to have other skin AEs, e.g., “itch” and “rash.” Such a connection is likely partly the result of the relatedness of the disease features and partly of the non-independence of the way that the medical care providers express themselves in the clinical narratives. Unexpected correlations can also be observed, e.g., the correlations in the large network between “auditory hallucination” and “vomiting,” and in the small separate networks between “kidney problem” and “asthma,” and “knee damage” and “increase liver function tests.”

To a certain extent, the co-occurrence analysis also stratifies the patients. Patients with AEs that are strongly associated have similar AE profiles and could be considered as a group. The characteristics of such groups of patients can be further examined in terms of the drugs they have received (polypharmacy may be the underlying cause), the diagnoses they have, their age and sex, their genetic variants, their proteome expression etc., in order to determine the underlying cause for why they are affected by both AEs. This might identify risk factors for the generation of the AEs in question.

#### Weighted edges network

**Figure 9:**
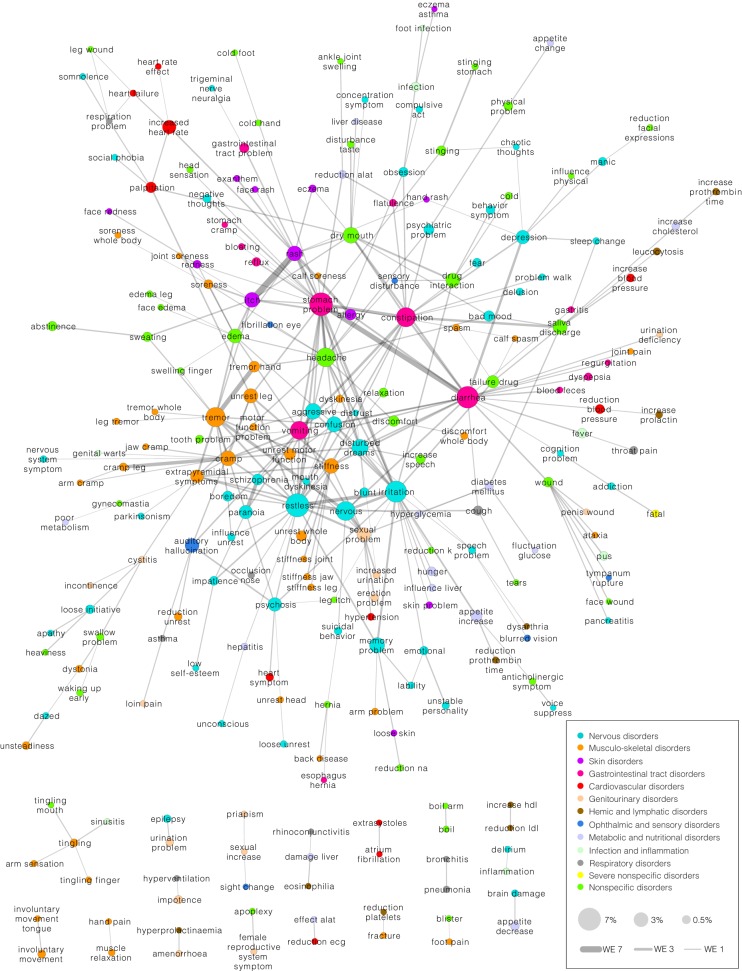
**Network of adverse event (AE) associations based on which patients the AEs affect**. Shown are only the significant weighted edges as determined by extracting the multiscale backbone at a significance level of 0.01. In this network, there are 240 AEs and 327 edges. The nodes are colored according to the anatomical class they belong to. Node size reflects the fraction of patients out of the entire patient population that are affected by a given AE. The 10 most prevalent AEs have been taken out of the network to allow better visualization.

Another AE co-occurrence network was created as shown in Figure 9 when exploring the co-occurrences of AEs in patients by assigning weighted edges and extracting the multiscale backbone at a significance level of 0.01. Ten common AEs were removed (the ten most prevalent AEs at the mental health center), which yielded a network of 240 AEs and 327 associations. Hence there is a ratio of 1.36 associations to each AE, which is not far from the ratio of the co-occurrence score network (1.51). The two networks share 108 AEs (62% of the nodes from the co-occurrence score network) and 189 associations (72% of the associations in the co-occurrence score network). Considering that there are 1190 AEs and 707455 possible AE associations, these percentages are high. Thus, in spite of some differences in the networks, the methods do yield very similar results.

Comparable to the network of Figure 8, the network in Figure 9 contains one large interconnected network and a number of smaller clusters (18 clusters) separated from the large network. Again, AEs related to the same anatomical area tend to gather in the same regions of the network. The AEs that one would expect to correlate do indeed co-occur in the same patients such as the association between “itch” and “rash.” Some of the associations seen in the network of Figure 8 reappear in the network of Figure 9, for example the association between “memory problem” and “arm problem” and between “saliva discharge” and “leucocytosis.” Furthermore, a number of other potentially interesting AE co-occurrences can be observed such as the associations between “trigeminal nerve neuralgia” and “gastrointestinal tract problem,” between “cramp” and “genital warts,” and between “rhinoconjunctivitis” and “damage liver.”

## Discussion

We have demonstrated a practical non-hypothesis driven approach to assess AE occurrences in patients. The presented methods exploit phenotypic AE data contained in EMRs, extracted as previously described (Eriksson et al., 2013, 2014), by clustering and visualizing data to enhance understanding of the AEs. The AE data is used to divide a cohort into patient groups, which allows us to investigate patterns in AE occurrence. While the results from the AE co-occurrence analysis show for the most part known and unsurprising correlations, unexpected correlations are also observed. Hence, an advantage of this method is that it allows the discovery of findings irrespectively of previous knowledge. It works from the entire space of AEs for all drugs in a concerted manner. Such findings may provide clues to further research that will help to enlighten the etiology of ADR genesis and understand in particular, which patients are affected. Especially associations between well-studied ADRs and less studied ADRs may provide insight to the less studied ADRs.

While extracting AEs from clinical narratives shows great promise, the results of the visualization presented here depend largely on the data quality. One has to keep in mind that the information contained in EMRs was not generated primarily for research purposes. This entails that symptoms that have not been clinically confirmed are in some cases also considered an AE if they are related to drug use. This may capture false positive AEs signals but also ensures that all signals are captured. Moreover, with regard to the data, the results are domain-specific as they depend on the prevalence of ADRs in the patient population being analyzed. Hence, the results showed here represent the AEs in a psychiatric cohort and employing these methods on other data sets will yield different results. Furthermore, every distance measure and statistical filtering mechanism has a bias and the methods that we have chosen is no exception. This is why two methods were employed to provide critical comparison, and though the networks are different the two methods still provide comparable results and are equally employable. They further complement each other by identifying different unexpected co-occurrences of AEs.

The data set used here is small for pharmacovigilance studies, and does not provide sufficient statistical evidence to conclude the existence of true correlations. However, the results still provide observations for further study and validate the methods for use in larger populations. The next step to complement the obtained results is to investigate the underlying causes for observed AE generation and correlations. The genomic or proteomic profiles of the patients might reveal explanations for the observed patterns. The root cause may also be the dosage of drug intake or polypharmacy.

Complex pathways and mechanisms of action of drugs determine ADR generation. Better understanding of drug responses and patient profiles can guide treatment procedures. Here we provide a starting point for further in depth analyses that increase the knowledge of AE-drug causality by revealing predisposing factors such as disease state, polypharmacy, or other co-occurring AEs. Ultimately, such knowledge may help prevent ADRs.

### Conflict of interest statement

The authors declare that the research was conducted in the absence of any commercial or financial relationships that could be construed as a potential conflict of interest.

## Abbreviations

ADR: Adverse Drug Reaction
AE: Adverse Event
ATC: Anatomical Therapeutic Chemical
EMR: Electronic Medical Record
ICD10: International Classification of Diseases, 10th revision
Tf-idf: term-frequency inverse document frequency.
